# The complete mitochondrial genome of *Aquila nipalensis* and its phylogenetic position

**DOI:** 10.1080/23802359.2019.1623118

**Published:** 2019-07-10

**Authors:** Chuang Zhou, Hongmei Tu, Yingzhu Chen, Liang Dou, Yang Meng, Nan Yang, Bisong Yue, Yongjie Wu

**Affiliations:** a Key Laboratory of Bioresources and Ecoenvironment, Ministry of Education, College of Life Sciences, Sichuan University, Chengdu, P. R. China;; b Institute of Qinghai-Tibetan Plateau, Southwest Minzu University, Chengdu, P. R. China

**Keywords:** *Aquila nipalensis*, mitochondrial genome, phylogenetic analysis

## Abstract

Mitochondrial genome sequences are valuable resources for systematics and conservation biology studies. In this paper, we present the complete mitogenome of *Aquila nipalensis* which was 18,450 bp in length. The gene content and arrangement were typical for avian mtDNA. The overall A + T content of was 54.1%, and the AT skew was calculated as 0.12 for the complete mitogenome of *A. nipalensis*. The maximum-likelihood (ML) tree based on the concatenated 12 protein-coding genes (PCGs) revealed the basal phylogenetic position of *A. nipalensis* in *Aquila*.

Major declines in the steppe eagle (*Aquila nipalensis*) population have been reported, and potential threats to *A. nipalensis* consist of habitat loss, human persecution, and electrocution on power lines (Meyburg et al. 2012). The mitochondrial genome is informative and has been widely employed in systematics and conservation biology studies (Curole and Kocher 1999). In this study, we first sequenced and analyzed the complete mitochondrial genome of *A. nipalensis*. The mitogenome of *A. nipalensis* could provide molecular data for the study of avian evolutionary history and provide new insights into the conservation strategy.

Muscle sample of a wild *A. nipalensis* that died of airport protection facility for bird strikes was collected from Aba Hongyuan Airport (102°21′24″, 32°31′53″). The specimen was preserved in the Museum of Sichuan University now. Some primers used in this study were obtained from previous study (Amer et al. 2013) and others were designed on the basis of the acquired sequences. Genomic DNA extraction, PCR amplification, sequencing and annotation were performed according to the methods described by Zhou et al. (2017).

The complete mitogenome of *A. nipalensis* (GeneBank accession number MK860035) was 18,450 bp in length, which composed of 13 protein-coding (PCGs), two ribosomal RNA (rRNA), 22 transfer RNA (tRNA) genes, and one control region. All the genes of *A. nipalensis* encoded on the H-strand with the exception of one PCG (ND6) and eight tRNAs (tRNA^Gln^, tRNA^Ala^, tRNA^Asn^, tRNA^Cys^, tRNA^Tyr^, tRNA^Ser(UCN)^, tRNA^Pro^, and tRNA^Glu^), which was typical for avian mtDNA (Dove et al., 2008). Overall, nucleotide base composition of *A. nipalensis* mitogenome was 30.3% A, 32.0% C, 13.9% G, and 23.8% T with an overall A + T content of 54.1%. Meanwhile, the AT skew was calculated as 0.12 for the complete mitogenome of *A. nipalensis*.

To determine the taxonomic status of *A. nipalensis*, we performed the phylogenetic analysis on the basis of the concatenated 12 PCGs using RAxML (Stamatakis 2014) (Figure 1). Phylogenetic tree showed that *A. nipalensis* possessed a basal phylogenetic position in *Aquila*. In conclusion, our study described the complete mitogenome of *A. nipalensis*, and defined its phylogenetic position, which would facilitate further investigations of molecular evolution and conservation of this species.

**Figure 1.**
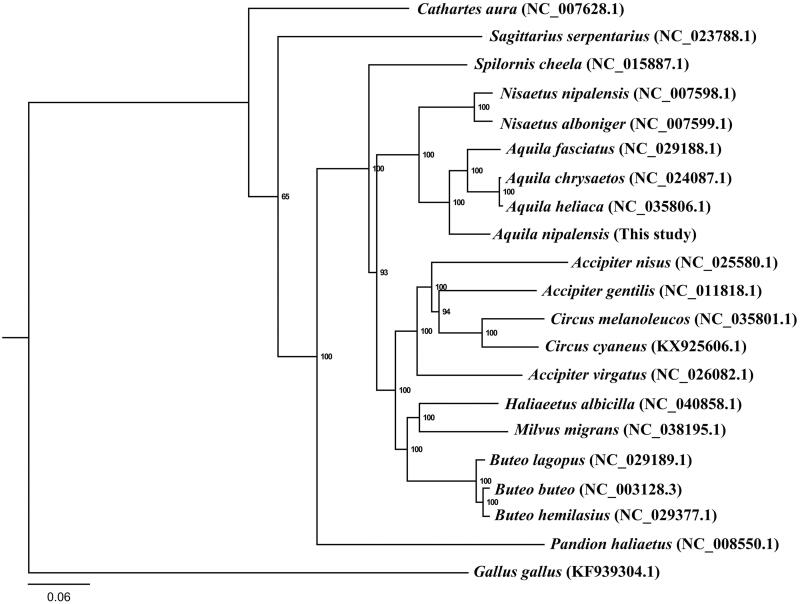
Phylogenetic tree of *A. nipalensis* based on the maximum-likelihood (ML) analysis of 12 concatenated mitochondrial protein-coding genes (with the exception of ND6). The bootstrap values for the ML analysis are shown on the nodes.
